# Supplementary material for: Pharmacologic neuroprotective agents for the treatment of perinatal asphyxia in low-income and lower-middle-income countries: A systematic review and meta-analysis of randomised controlled trials
Source: PLoS One. 2025 Dec 4;20(12):e0337798. doi: 10.1371/journal.pone.0337798 (PMC12677539; doi:10.1371/journal.pone.0337798)
Supplement: S2 File — (DOCX) [file pone.0337798.s002.docx]

**S2 – Search Strategy**

**PUBMED Search Strategy/Trail**

| S/N | Keywords | Search Strategy (27-August-2024) | Results |
| --- | --- | --- | --- |
| #1 | Pharmacologic neuroprotective | (((((((((((neuroprotect*[Title/Abstract]) OR (erythropoietin[Title/Abstract])) OR (allopurinol[Title/Abstract])) OR (N-acetyl cysteine[Title/Abstract])) OR (acetylcysteine[Title/Abstract])) OR (antioxidant[Title/Abstract])) OR (NAC[Title/Abstract])) OR ("magnesium sulphate"[Title/Abstract])) OR (melatonin[Title/Abstract])) OR (xenon[Title/Abstract])) OR (helium[Title/Abstract])) OR (anti-inflammatory) | 1,245,768 |
| #2 | Perinatal asphyxia | (((("perinatal asphyxia"[Title/Abstract]) OR ("neonatal encephalopathy"[Title/Abstract])) OR ("birth asphyxia"[Title/Abstract])) OR ("hypoxic-ischemic encephalopathy"[Title/Abstract])) OR ("neonatal asphyxia"[Title/Abstract]) | 10,703 |
| #3 | #1 AND #2 | ((((("perinatal asphyxia"[Title/Abstract]) OR ("neonatal encephalopathy"[Title/Abstract])) OR ("birth asphyxia"[Title/Abstract])) OR ("hypoxic-ischemic encephalopathy"[Title/Abstract])) OR ("neonatal asphyxia"[Title/Abstract])) AND ((((((((((((neuroprotect*[Title/Abstract]) OR (erythropoietin[Title/Abstract])) OR (allopurinol[Title/Abstract])) OR (N-acetyl cysteine[Title/Abstract])) OR (acetylcysteine[Title/Abstract])) OR (antioxidant[Title/Abstract])) OR (NAC[Title/Abstract])) OR ("magnesium sulphate"[Title/Abstract])) OR (melatonin[Title/Abstract])) OR (xenon[Title/Abstract])) OR (helium[Title/Abstract])) OR (anti-inflammatory)) | 1,438 |
| #4 | Filter 2004-2024 | ((((("perinatal asphyxia"[Title/Abstract]) OR ("neonatal encephalopathy"[Title/Abstract])) OR ("birth asphyxia"[Title/Abstract])) OR ("hypoxic-ischemic encephalopathy"[Title/Abstract])) OR ("neonatal asphyxia"[Title/Abstract])) AND ((((((((((((neuroprotect*[Title/Abstract]) OR (erythropoietin[Title/Abstract])) OR (allopurinol[Title/Abstract])) OR (N-acetyl cysteine[Title/Abstract])) OR (acetylcysteine[Title/Abstract])) OR (antioxidant[Title/Abstract])) OR (NAC[Title/Abstract])) OR ("magnesium sulphate"[Title/Abstract])) OR (melatonin[Title/Abstract])) OR (xenon[Title/Abstract])) OR (helium[Title/Abstract])) OR (anti-inflammatory)) Filters: from 2004 - 2024 | 1,332 |

WEB OF SCIENCE

| **Search** | **Search terms** | **Search strategy** | **Results** |
| --- | --- | --- | --- |
| #4 | **Filter** | **#1 AND #2 AND #3**  **Timespan: 2004-01-01 to 2024-12-31 (Publication Date)** | [692](http://webofscience-clarivate-cn-s.webvpn.njmu.edu.cn:8118/wos/alldb/summary/bf991e66-8d9f-46fe-8d22-93f82088373f-0104253654/relevance/1) |
| #3 |  | **#1 AND #2** | [744](http://webofscience-clarivate-cn-s.webvpn.njmu.edu.cn:8118/wos/alldb/summary/f28ce529-03b3-4e78-ac1f-8567d602856f-01042532f1/relevance/1) |
| #2 | **Perinatal asphyxia** | **TS=(“perinatal asphyxia” OR “neonatal encephalopathy" OR "birth asphyxia" OR "hypoxic-ischemic encephalopathy" OR "neonatal asphyxia”)** and **Preprint Citation Index** (Exclude – Database) | [16,925](http://webofscience-clarivate-cn-s.webvpn.njmu.edu.cn:8118/wos/alldb/summary/3da0d358-bb32-4b83-81c3-7f4226f1a1bc-0104253277/relevance/1) |
| #1 | **Pharmacologic neuroprotective** | **TS=(Neuroprotect*OR Erythropoietin OR Allopurinol OR N-acetyl cysteine OR Acetylcysteine OR Antioxidant OR NAC OR "magnesium sulphate" OR Melatonin OR Xenon OR Helium OR "anti-inflammatory")** and **Preprint Citation Index** (Exclude – Database) | [1,538,126](http://webofscience-clarivate-cn-s.webvpn.njmu.edu.cn:8118/wos/alldb/summary/61471a5f-ddc7-4100-af2f-8e638de321da-0104252e21/relevance/1) |

CINAHL

| **Search** | **Search terms** | **Search strategy** | **Results** |
| --- | --- | --- | --- |
| S4 | **Filter** | **S1 AND S2**  **Limiters - Publication Date: 20040101-20241231** | (145) |
| S3 |  | **S1 AND S2** | (152) |
| S2 | **Perinatal asphyxia** | “perinatal asphyxia” OR “neonatal encephalopathy" OR "birth asphyxia" OR "hypoxic-ischemic encephalopathy" OR "neonatal asphyxia” | (3,993) |
| S1 | **Pharmacologic neuroprotective** | Neuroprotect*OR Erythropoietin OR Allopurinol OR N-acetyl cysteine OR Acetylcysteine OR Antioxidant OR NAC OR "magnesium sulphate" OR Melatonin OR Xenon OR Helium OR "anti-inflammatory" | (81,899) |
