# Supplementary material for: Pharmacologic neuroprotective agents for the treatment of perinatal asphyxia in low-income and lower-middle-income countries: A systematic review and meta-analysis of randomised controlled trials
Source: PLoS One. 2025 Dec 4;20(12):e0337798. doi: 10.1371/journal.pone.0337798 (PMC12677539; doi:10.1371/journal.pone.0337798)
Supplement: S5 File — (DOCX) [file pone.0337798.s005.docx]

**S5 – Funnel plots for Outcomes used in the Meta-analysis**
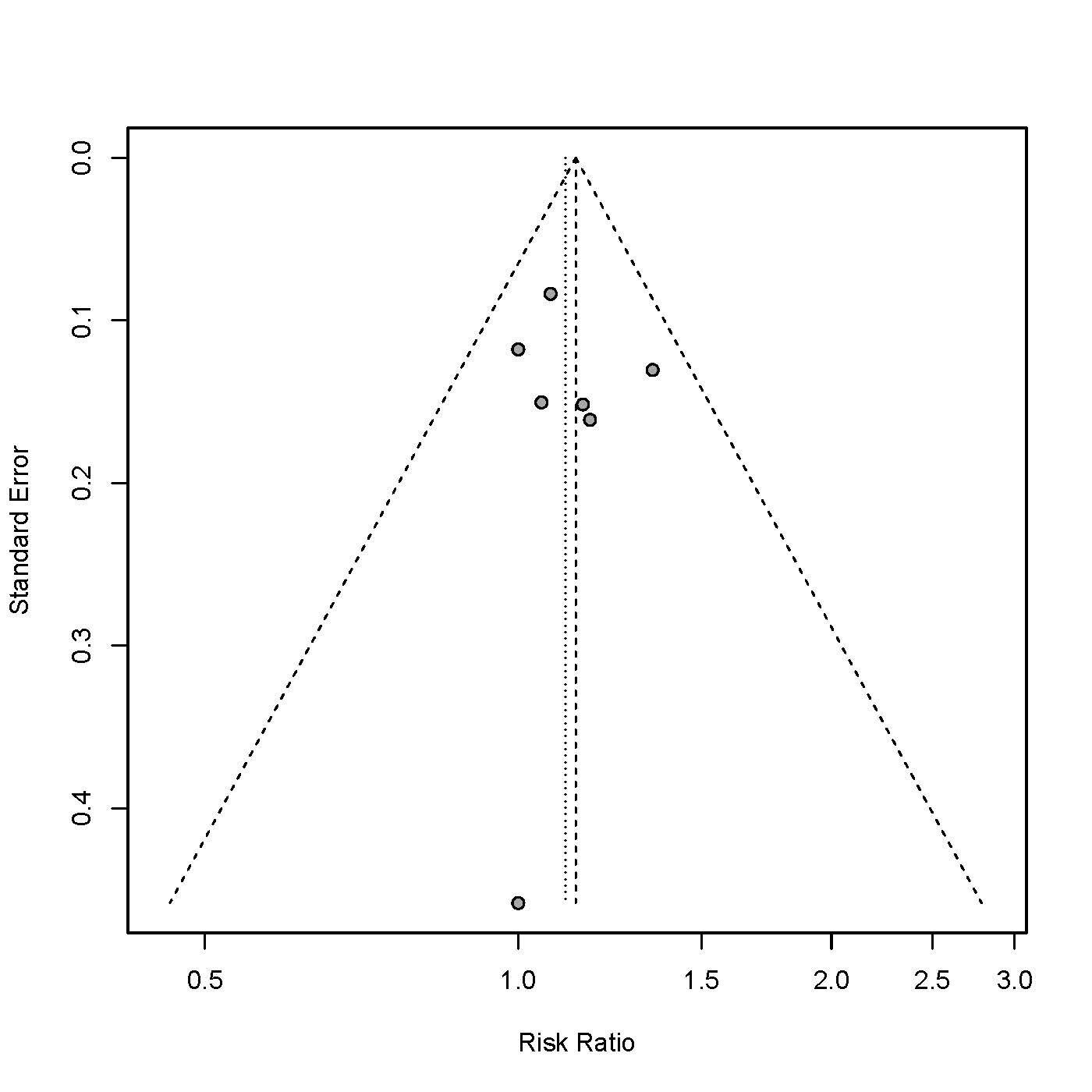


**Funnel Plot 1 – Effect of the neuroprotective agents on survival**


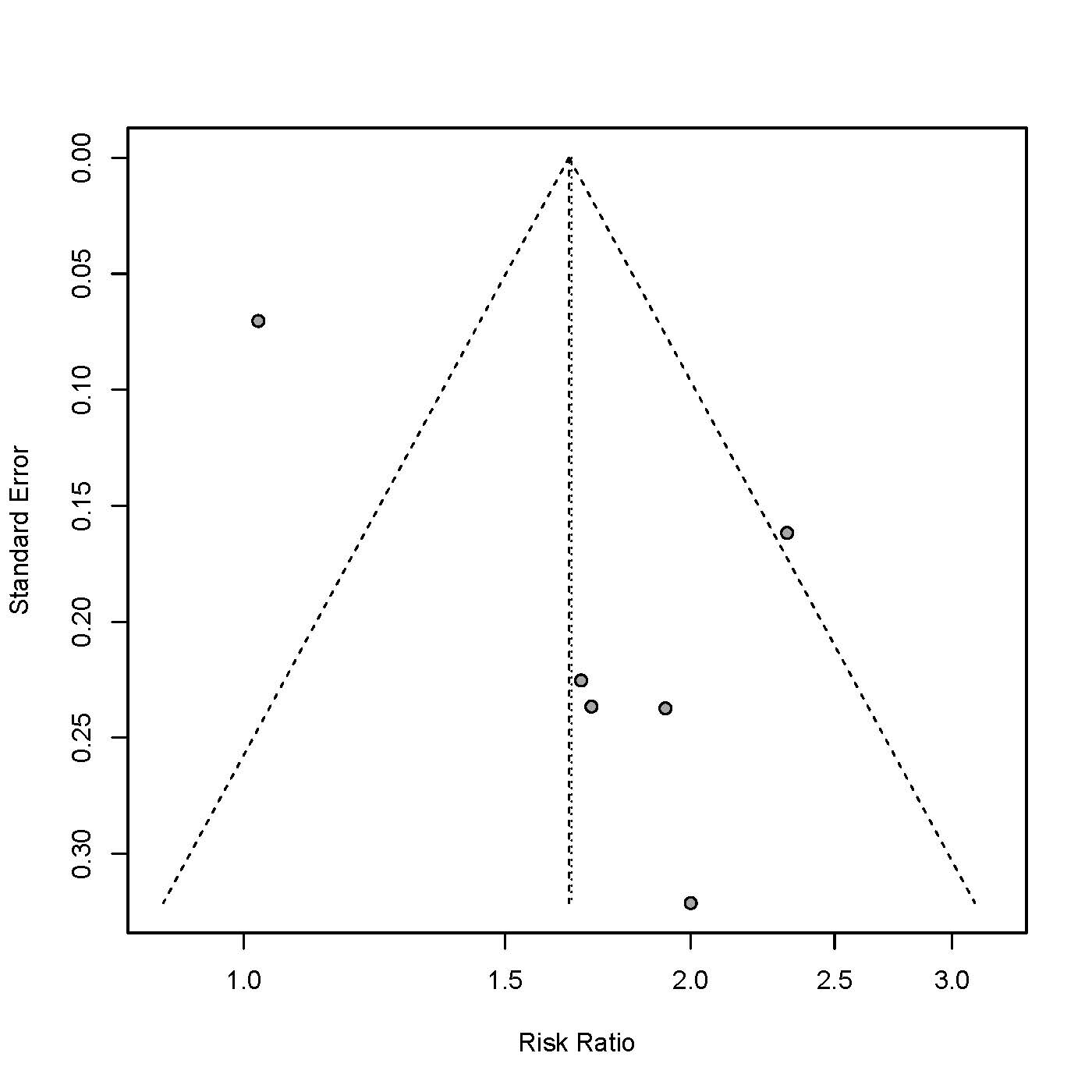


**Funnel Plot 2 – Effect of the neuroprotective agents on the successful initiation of oral feeds at discharge**


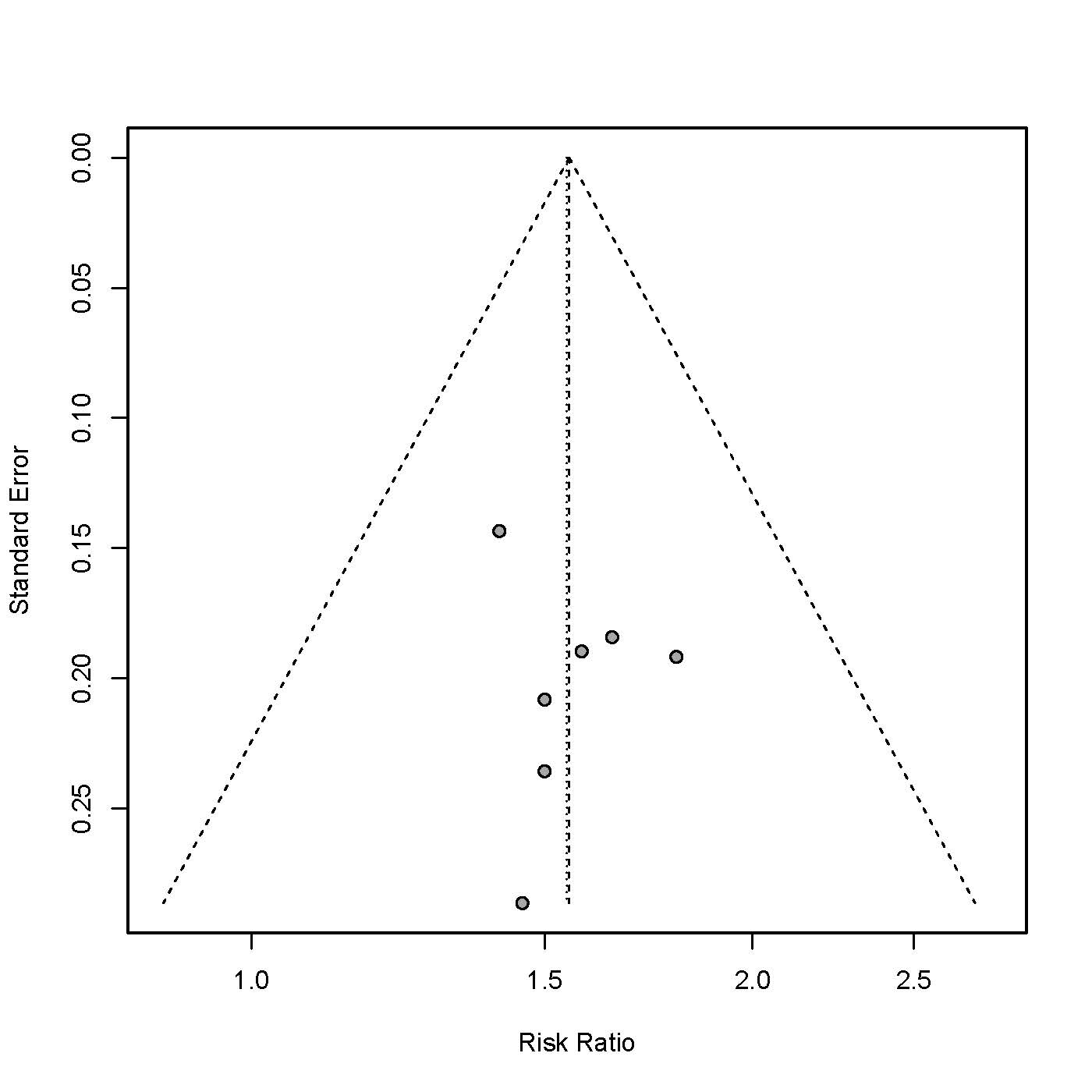


**Funnel Plot 3 – Effect of the neuroprotective agents on abnormal Neuroimaging features at discharge/one month**


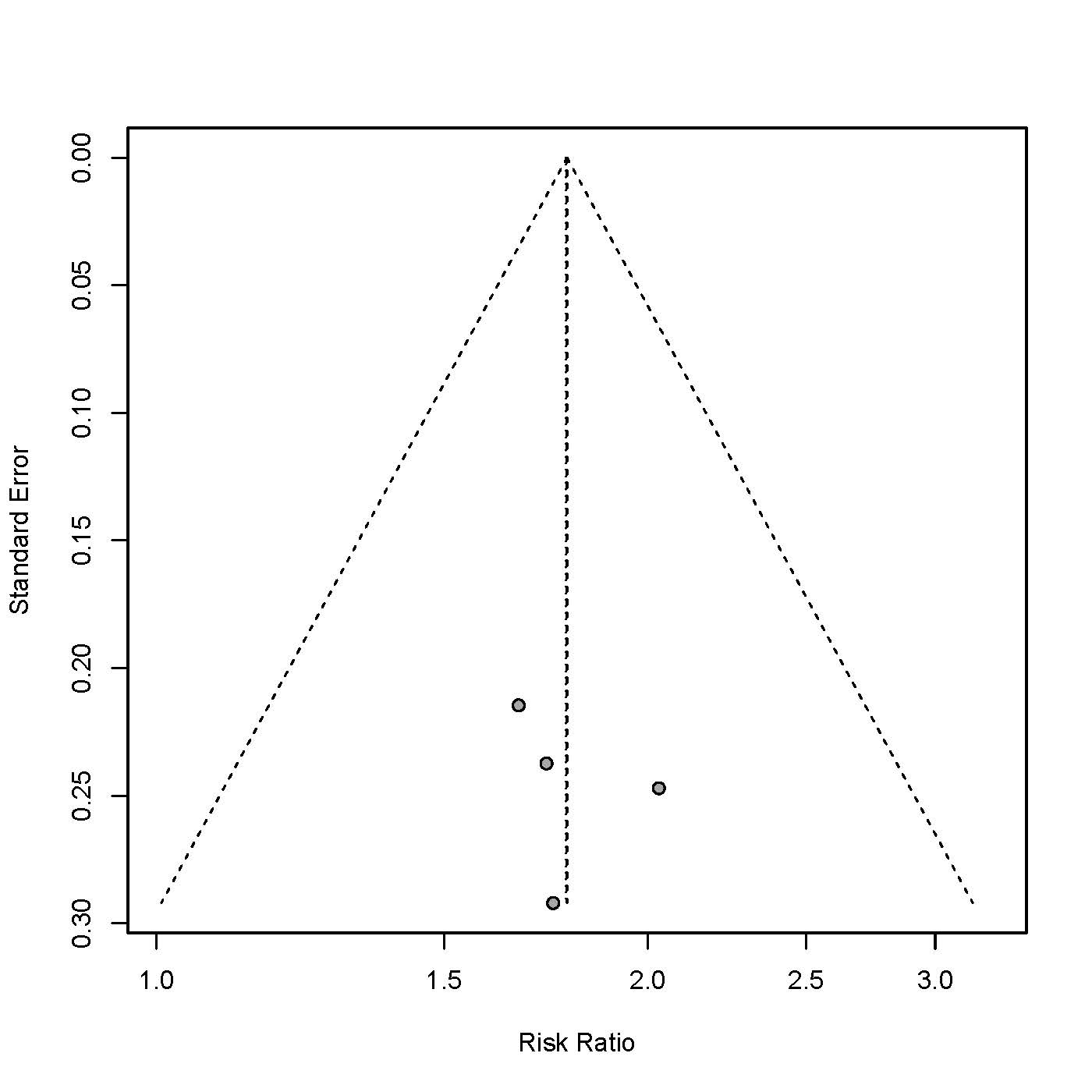


**Funnel Plot 4 – Effect the neuroprotective agents on abnormal neurologic features at discharge/one month**


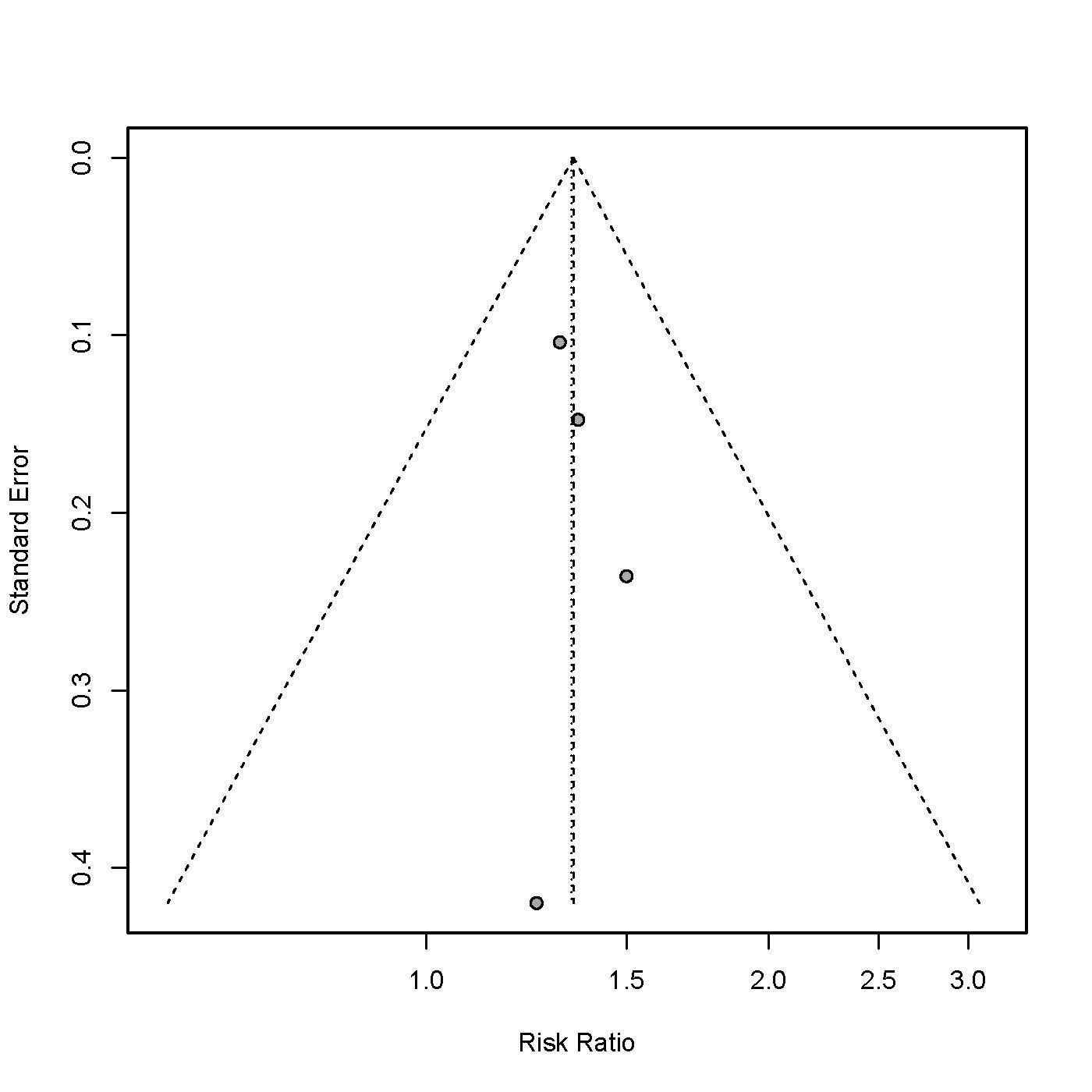


**Funnel Plot 5 – Effect of the neuroprotective agents on the need for antiepileptic drug at discharge**
