# Supplementary material for: Pharmacologic neuroprotective agents for the treatment of perinatal asphyxia in low-income and lower-middle-income countries: A systematic review and meta-analysis of randomised controlled trials
Source: PLoS One. 2025 Dec 4;20(12):e0337798. doi: 10.1371/journal.pone.0337798 (PMC12677539; doi:10.1371/journal.pone.0337798)
Supplement: S6 File — (DOCX) [file pone.0337798.s006.docx]

**S6 – Studies that appear to meet the inclusion criteria, but excluded**^1–32^

Studies 1,2,5,7,12,13,14,15,16,18,19,20,22,23,24,27,28,31,32 were not Randomised controlled trials.

Studies 2 and 21 had controls which were not placebo.

Study 8 had therapeutic hypothermia included in the standard treatment, so the neuroprotective agent was given as an adjunct. The use of therapeutic hypothermia was an exclusion criteria in this review.

Studies 3, 6, 9, 10 and 17 did not describe what the routine/standard care in the units were, so it was unclear if therapeutic hypothermia or other neuroprotective agents were in standard use.

Studies 28 and 29 were not carried out in LILMICs.

Studies 11 and 24 had no statement about the use of standard/routine care in the neonates.

1. Pan JJ, Wu Y, Liu Y, Cheng R, Chen XQ, Yang Y. The effect of erythropoietin on neonatal hypoxic-ischemic encephalopathy: An updated meta-analysis of randomized control trials. *Front Pediatr* 2023; 10: 1074287. DOI:10.3389/FPED.2022.1074287.

2. El-Shafie A, El Lahouny D, El Latif Omar Z, Hamoud FA. Neuroprotectors in hypoxic ischemic encephalopathy in neonates: a systematic review. *Menoufia Medical Journal* 2021; 34: 413. DOI:10.4103/mmj.mmj_330_19.

3. Gathwala G, Marwah A, Gahlaut V, Marwah P. *Effect of High-dose Phenobarbital on Oxidative Stress in Perinatal Asphyxia: An Open Label Randomized Controlled Trial*. 2011.

4. Khan MH, Ann Q, Khan MS, Ahmad N, Ahmed M. Efficacy of Magnesium Sulfate in Addition to Melatonin Therapy in Neonates With Hypoxic-Ischemic Encephalopathy. *Cureus*. Epub ahead of print 12 January 2022. DOI: 10.7759/cureus.21163. DOI:10.7759/cureus.21163.

5. Marsia S, Kumar D, Raheel H, Salman A, Aslam B, Ikram A, et al. Evaluating the Safety and Efficacy of Erythropoietin Therapy for Neonatal Hypoxic-Ischemic Encephalopathy: A Systematic Review and Meta-Analysis. *Pediatric Neurology* 2024; 152: 4–10. DOI:10.1016/j.pediatrneurol.2023.12.008.

6. Nanda AK, Jalan A, Pradhan SK, Kumar TVR, Pradhan PC. Effect of magnesium sulphate infusion on neonatal outcomes in babies with perinatal asphyxia. *Int J Health Sci (Qassim)* 2022; 6: 13064–13075. DOI:10.53730/IJHS.V6NS2.8450.

7. Pius S, Bello M, Ambe JP, Machoko Y, Clement AY, Genesis R, et al. Pilot Study: Magnesium Sulphate Administration and Early Resolution of Hypoxic Ischemic Encephalopathy in Severe Perinatal Asphyxia. *Open J Pediatr* 2019; 09: 89–102. DOI:10.4236/ojped.2019.91009.

8. Wu YW, Comstock BA, Gonzalez FF, Mayock DE, Goodman AM, Maitre NL, et al. Trial of Erythropoietin for Hypoxic–Ischemic Encephalopathy in Newborns. *New England Journal of Medicine* 2022; 387: 148–159. DOI:10.1056/nejmoa2119660.

9. Zhu C, Kang W, Xu F, Cheng X, Zhang Z, Jia L, et al. Erythropoietin improved neurologic outcomes in newborns with hypoxic-ischemic encephalopathy. *Pediatrics*; 124. Epub ahead of print August 2009. DOI: 10.1542/peds.2008-3553. DOI:10.1542/peds.2008-3553.

10. Benders MJNL, Bos AF, Rademaker CMA, Rijken M, Torrance HL, Groenendaal F, et al. Early postnatal allopurinol does not improve short term outcome after severe birth asphyxia. *Arch Dis Child Fetal Neonatal Ed*; 91. Epub ahead of print May 2006. DOI: 10.1136/adc.2005.086652. DOI:10.1136/adc.2005.086652.

11. Gunes T, Ozturk MA, Koklu E, Kose K, Gunes I. Effect of Allopurinol Supplementation on Nitric Oxide Levels in Asphyxiated Newborns. *Pediatr Neurol* 2007; 36: 17–24. DOI:10.1016/j.pediatrneurol.2006.08.005.

12. Kaandorp JJ, Van Bel F, Veen S, Derks JB, Groenendaal F, Rijken M, et al. Long-term neuroprotective effects of allopurinol after moderate perinatal asphyxia: Follow-up of two randomised controlled trials. *Arch Dis Child Fetal Neonatal Ed*; 97. Epub ahead of print May 2012. DOI: 10.1136/archdischild-2011-300356. DOI:10.1136/archdischild-2011-300356.

13. Pius S, Bello M, Ambe JP, Yenti M, Genesis R, Clement AY, et al. Magnesium Sulphate Treated Severely Asphyxiated Neonates, Their Characteristic and Outcome. *Asian Journal of Pediatric Research* 2019; 1–9. DOI:10.9734/ajpr/2018/v1i230092.

14. Robertson NJ, Tan S, Groenendaal F, Van Bel F, Juul SE, Bennet L, et al. Which neuroprotective agents are ready for bench to bedside translation in the newborn infant? *Journal of Pediatrics*; 160. Epub ahead of print 2012. DOI: 10.1016/j.jpeds.2011.12.052. DOI:10.1016/j.jpeds.2011.12.052.

15. Sengupta V. Article title: A SYSTEMATIC REVIEW TO DETERMINE THE ROLE OF ERYTHROPOIETIN IN PREVENTION OF HYPOXIC ISCHAEMIC ENCEPHALOPATHY OF NEWBORN A SYSTEMATIC REVIEW TO DETERMINE THE ROLE OF ERYTHROPOIETIN IN PREVENTION OF HYPOXIC ISCHAEMIC ENCEPHALOPATHY OF NEWBORN. DOI: 10.14293/PR2199.000540.v1. DOI:10.14293/PR2199.000540.v1.

16. Shepherd E, Karim T, McIntyre S, Goldsmith S, Keir A, Badawi N, et al. Neonatal magnesium sulphate for neuroprotection: A systematic review and meta-analysis. *Developmental Medicine and Child Neurology*. Epub ahead of print 1 September 2024. DOI: 10.1111/dmcn.15899. DOI:10.1111/dmcn.15899.

17. Singh D, Kumar P, Narang A. A randomized controlled trial of phenobarbital in neonates with hypoxic ischemic encephalopathy. *Journal of Maternal-Fetal and Neonatal Medicine* 2005; 18: 391–395. DOI:10.1080/13895260500327979.

18. Tagin M, Shah PS, Lee KS. Magnesium for newborns with hypoxic-ischemic encephalopathy: A systematic review and meta-analysis. *Journal of Perinatology* 2013; 33: 663–669. DOI:10.1038/jp.2013.65.

19. Yang G, Xue Z, Zhao Y. Efficacy of erythropoietin alone in treatment of neonates with hypoxic-ischemic encephalopathy A protocol for systematic review and meta-analysis. Epub ahead of print 2021. DOI: 10.17605/OSF.IO/FERUS. DOI:10.17605/OSF.IO/FERUS.

20. Ivain P, Montaldo P, Khan A, Elagovan R, Burgod C, Morales MM, et al. Erythropoietin monotherapy for neuroprotection after neonatal encephalopathy in low-to-middle income countries: a systematic review and meta-analysis. *Journal of Perinatology* 2021; 41: 2134–2140. DOI:10.1038/s41372-021-01132-4.

21. El Farargy MS, Soliman NA. A randomized controlled trial on the use of magnesium sulfate and melatonin in neonatal hypoxic ischemic encephalopathy. *J Neonatal Perinatal Med* 2020; 12: 379–384. DOI:10.3233/NPM-181830.

22. Razak A, Hussain A. Erythropoietin in perinatal hypoxic-ischemic encephalopathy: A systematic review and meta-analysis. *J Perinat Med* 2019; 47: 478–489. DOI:10.1515/jpm-2018-0360.

23. Merigo G, Florio G, Madotto F, Magliocca A, Silvestri I, Fumagalli F, et al. Treatment with inhaled Argon: a systematic review of pre-clinical and clinical studies with meta-analysis on neuroprotective effect. *EBioMedicine*; 103. Epub ahead of print 30 April 2024. DOI: 10.17605/OSF.IO/7983D. DOI:10.17605/OSF.IO/7983D.

24. Gathwala G, Khera A, Singh J, Balhara B. Magnesium for Neuroprotection in birth asphyxia. *J Pediatr Neurosci* 2010; 5: 102–104.

25. Sreenivasa B, Lokeshwari K, Joseph N. Role of magnesium sulphate in management and complications of birth asphyxia. *Sri Lanka Journal of Child Health* 2017; 46: 148–151.

26. Zen Lee CY, Chakranon P, Huey Lee SW. Comparative efficacy and safety of neuroprotective therapies for neonates with hypoxic ischemic encephalopathy: A network meta-analysis. *Frontiers in Pharmacology*; 10. Epub ahead of print 2019. DOI: 10.3389/fphar.2019.01221. DOI:10.3389/fphar.2019.01221.

27. Liu TS, Yin ZH, Yang ZH, Wanf LN. The effects of monotherapy with erythropoietin in neonatal hypoxic-ischemic encephalopathy on neurobehavioral development: a systematic review and meta-analysis. *Eur Rev Med Pharmacol Sci* 2021; 25: 2318–2326.

28. Jing S, Ying X, Dezhi M. Clinical evidence of pharmacological treatment of neonatal hypoxic-ischemic encephalopathy. *Chinese Journal of Contemporary Pediatrics*; 1.

29. Ying-Juan W, Kai-Li P, Xiao-Li Z, Huan Q, Sheng-Quan C. Therapeutic effects of erythropoietin on hypoxic-ischemic encephalopathy. *Chinese Journal of Contemporary Pediatrics*; 13.

30. Garg BD, Sharma D, Bansal A. Systematic review seeking erythropoietin role for neuroprotection in neonates with hypoxic ischemic encephalopathy: presently where do we stand. *Journal of Maternal-Fetal and Neonatal Medicine* 2018; 31: 3214–3224. DOI:10.1080/14767058.2017.1366982.

31. Chaudhari T, McGuire W. Allopurinol for preventing mortality and morbidity in newborn infants with suspected hypoxic-ischaemic encephalopathy. *Cochrane Database of Systematic Reviews*. Epub ahead of print 2008. DOI: 10.1002/14651858.CD006817.pub2. DOI:10.1002/14651858.CD006817.pub2.

32. Gowda BB, Rath C, Muthusamy S, Nagarajan L, Rao S. Outcomes of Neonates with Hypoxic-Ischemic Encephalopathy Treated with Magnesium Sulfate: A Systematic Review with Meta-analysis. *The Journal of Peditrics*. Epub ahead of print November 2023. DOI: 10.17605/OSF.IO/FRM4D. DOI:10.17605/OSF.IO/FRM4D.
