# Supplementary material for: Pharmacologic neuroprotective agents for the treatment of perinatal asphyxia in low-income and lower-middle-income countries: A systematic review and meta-analysis of randomised controlled trials
Source: PLoS One. 2025 Dec 4;20(12):e0337798. doi: 10.1371/journal.pone.0337798 (PMC12677539; doi:10.1371/journal.pone.0337798)
Supplement: Attachment — Submitted filename: Response to Reviewers.pdf [file pone.0337798.s009.pdf]

Department of Paediatrics,
Babcock University,
Ilishan-Remo,
Ogun State,
Nigeria.
15th October 2025

The Editor-in-Chief,
PLOS ONE

Dear Sir/Ma,

LETTER OF RESPONSE TO REVIEWERS

I am writing our rebuttal to the reviewers' comments on the systematic review titled '**Pharmacologic neuroprotective agents for the treatment of perinatal asphyxia in low-income and lower-middle-income countries: a systematic review and meta-analysis of randomised controlled trials**'.

1. Statistical part should be clarified whether a statistician was a part of the team.
   Yes, one of the authors is indeed a Statistician and she was involved with the process of the systematic review from the beginning, through the meta-analysis and up till submission: Olunike Rebecca Abodunrin. The appropriate statistical methods were used and verified by the statistician on the team.
2. Why are there two different abbreviations used since I believe there could be only one: LMIC and LILMIC?
   The review focused on low-income and lower middle-income countries (LILMICs) rather than low- and middle-income countries (LMIC). We decided to focus on LILMICs for better homogeneity of the included countries, as it was known that upper middle-income countries (which are part of LMIC but not part of LILMIC) have distinctive qualities, which may not be shared by pure LILMIC. However, LMICs were mentioned in the Background aspects of the Manuscript at points where we were referring to previous work and publications, which focused on LMIC (which included our subject area of LILMIC). The appearances of LMICs from referenced texts were found on pages 2-4.

3. In the materials the Table 6 seems not in the appropriate form, it is not possible to read the text. Also, the references are missing, there is only minor part appearing at the end of the available materials.

   The font has been increased in size for both Tables 5 and 6, shown on pages 24-27

4. Line 33 on page 1, the spelling of “Biostatistics” was corrected.
5. Please state what role the funders took in the study.

   "The funders had no role in study design, data collection and analysis, decision to publish, or preparation of the manuscript."

6. Please include captions for your Supporting Information files at the end of your manuscript, and update any in-text citations to match accordingly.

   The captions for the Supporting Information files are now included at the end of the manuscript, and supplementary files 1-6 (S1 – S6) have been re-submitted.

7. Reviewer 2: Has the statistical analysis been performed appropriately and rigorously?

   Yes, one of the authors is indeed a Statistician and she was involved with the process of the systematic review from the beginning, through the meta-analysis and up till submission: Olunike Rebecca Abodunrin. The appropriate statistical methods were used and verified by the statistician on the team.

8. Reviewer 1: better keywords can be selected which are not a part of the title

   The keywords section has now been updated as follows: perinatal asphyxia, neonatal encephalopathy, pharmacologic neuroprotective agents, magnesium sulphate, neurodevelopmental outcomes, low-income and lower-middle-income countries

9. Reviewer1: forest plot for different outcomes have not been provided.

   These are shown in Figures 2 – 12.

10. Reviewer 1: Conclusion heading does not have complete text.

    This has been checked.

We sincerely appreciate your attention.

Yours sincerely,

Dr. Victor Ayodeji Ayeni
