# Supplementary material for: Pharmacologic neuroprotective agents for the treatment of perinatal asphyxia in low-income and lower-middle-income countries: A systematic review and meta-analysis of randomised controlled trials
Source: PLoS One. 2025 Dec 4;20(12):e0337798. doi: 10.1371/journal.pone.0337798 (PMC12677539; doi:10.1371/journal.pone.0337798)
Supplement: Attachment — Submitted filename: Review of Manuscript Number PONE.docx [file pone.0337798.s007.docx]

Review of Manuscript Number PONE-D-25-16916
"Pharmacologic neuroprotective agents for the treatment of perinatal asphyxia in low-income and lower-middle-income countries: a systematic review and meta-analysis of randomised controlled trials"

## The paper by Victor Ayodeji Ayeni et al is a systematic review and metanalysis of eligible randomised controlled trials (RCTs) published between 2000 and 2024 on various pharmacologic neuroprotective agents for the treatment of perinatal asphyxia compared with placebo or standard care, excluding therapeutic hypothermia, in LILMICs. Data on survival and neurodevelopmental outcomes are analysed. The article fulfils some of the criteria for publication: the authors claim it is an original work and the results reported have not been published elsewhere. In the aspect of statistical analysis, I would very much appreciate if the article could be subjected to an expert on the field. The conclusions are presented in an understandable and appropriate fashion and are supported by the given data. The article is is written in good intelligible English. The research meets the applicable standards for the ethics of experimentation and research integrity. The article adheres to appropriate reporting guidelines and community standards for data availability.

## Minor suggestions: why are there two different abbreviations used since I believe there could be only one: LMIC and LILMIC?

## In the materials the Table 6 seems not in the appropriate form, it is not possible to read the text. Also, the references are missing, there is only minor part appearing at the end of the available materials.
